# The Joint Association Between Multiple Dietary Patterns and Depressive Symptoms in Adults Aged 55 and Over in Northern China

**DOI:** 10.3389/fnut.2022.849384

**Published:** 2022-03-07

**Authors:** Yuxia Ma, Ruiqiang Li, Wenqiang Zhan, Xin Huang, Limin Zhang, Zhan Liu

**Affiliations:** ^1^Department of Nutrition and Food Hygiene, Hebei Province Key Laboratory of Environment and Human Health, School of Public Health, Hebei Medical University, Shijiazhuang, China; ^2^School of Public Health, Shanghai Jiao Tong University School of Medicine, Shanghai, China

**Keywords:** dietary patterns, depressive symptoms, elderly, quantile g-computation, Bayesian kernel machine regression

## Abstract

**Background:**

Depression is a common psychiatric disorder in older adults that affects their health-related quality of life. Two percent of adults over the age of 55 suffer from major depression, and the prevalence of depression increases with age. Even in the absence of major depressive disorder, 10–15% of older adults have clinically significant depressive symptoms.

**Objectives:**

Epidemiological studies on the association between different gender eating patterns and depression show inconsistent associations. Our study examined whether different gender eating patterns are related to depression. We consider eating patterns individually and as a joint exposure to predefined eating patterns.

**Methods:**

Principal component analysis (PCA) was performed on the data of the 24-h diet recall dietary intake, and the dietary pattern was determined. Linear regression models are used to explore the relationship between different diets and depression of men and women; weighted quantile sum (WQS) regression, quantile g calculation (qgcomp) and Bayesian kernel machine regression (BKMR) are performed as Secondary analysis.

**Results:**

In the dietary patterns model, we found that the vegetable and fruit-based diet has a significant inhibitory effect in women, and the egg-milk-based diet has a significant inhibitory effect in men. We found that when all dietary factors are above the 55th percentile, there is a significant positive correlation between multiple dietary patterns and depression risk. We also determined a positive correlation between meat and obesity risk and a negative correlation between egg and milk and vegetables and fruits.

**Conclusions:**

In the study population, after controlling for other baseline indicators and predictors of dietary pattern exposure, a fruit and vegetable-based diet was associated with a slightly healthier and lower risk of depression, while a meat-based dietary pattern associated with a higher risk of depression, and this association effect varies between genders.

## Introduction

Major depression is one of the most common, troublesome, and costly mental illness among adults worldwid (1). Meanwhile, major depression is the fourth leading cause of disability in the world, causing a serious socio-economic burden on a global scale (2). It is notable that depression has become a complex public health problem, and treatment responses vary greatly. The systemic complexity of depression, or the feedback process between the different drivers of the disorder, leads to the persistence of depression (3). Major depressive disorder (MDD) is a debilitating disease characterized by low mood, decreased interest, impaired cognitive function, and plant-based symptoms such as sleep or appetite disturbances. As time went on, depression shows great symptom heterogeneity from person to person, the incidence of MDD in women is approximately twice that of men, and affects one in six adults during their lifetime (4, 5). Moreover, Some studies have shown that depression in the elderly is a common mental illness that affects their health-related quality of life. Two percent of adults aged 55 or older suffer from major depression, and its prevalence increases with age. In addition, even in the absence of major depression, 10–15% of elderly people have clinically significant depressive symptoms (6).

At an early stage, evidence from laboratory, population studies, and clinical trials suggests that dietary patterns and specific dietary factors may influence depression risk (7). Recently, studies have shown that diet has a protective effect against depression (8). Higher intake of legume and vegetables and dairy products had a protective effect on the risk of depression (9). Diet quality has an effect on depression in adults and older adults. People with the worst diets were more likely to develop this mood disorder than those with the best diets. In addition, there is already research evidence that the consumption of food markers of an unhealthy diet and the consumption of soft drinks and processed fruit juices are associated with depression. This finding provides important evidence for the role of diet in depression, helping to prevent and treat this mood disorder (10). Many reviews confirm that similar dietary patterns of healthy eating are associated with reduced morbidity and all-cause mortality. Several potential mechanisms of this association have been discussed in other studies (11). The anti-inflammatory properties of foods in a healthy diet have been shown to affect the concentration of monoamines, which are believed to play a role in the regulation of mood and cognition. The antioxidant compounds in fruits and vegetables can reduce neuronal damage caused by oxidative stress, especially neurons in the hippocampus. Evidence also suggests that large amounts of long-chain omega-3 (n-3) polyunsaturated fatty acids (high in oily fish) can reduce the risk of depression. It may also be the cumulative effect of all these nutrients and their biochemical properties that affect the risk of depression (12).

With rapid economic growth and changes in lifestyles, China is undergoing a rapid epidemiological transition from infectious diseases to non-communicable diseases (NCD). Mental disorders such as depression are an important but often overlooked non-communicable disease, which is becoming an increasingly serious cause of disability, suicide and the burden of disease (13). A study in China showed that a vegetable-based diet was independently associated with a higher prevalence of depressive symptoms in older Chinese men (14). but plant-based eating patterns (vegan and vegetarian) were generally considered “healthy”, and has a wide range of health benefits (15). Moreover, fruits and vegetables as anti-inflammatory foods can reduce the inflammatory state to a certain extent and have a certain protective effect on depression (16). A previous meta-analysis reported that the combined prevalence of depressive symptoms in the elderly in China is 22.7%, and the prevalence of depressive symptoms in women (24.2%) is slightly higher than that of men (19.4%) (17). Although the number of people suffering from depression has increased in recent years, compared with other developed countries, there have been relatively few studies on depression in China (13). In cross-sectional and prospective studies, both the Mediterranean diet and the “anti-inflammatory” diet are associated with lowering the risk of depression (18). Therefore, it is worth exploring the relationship between dietary patterns and depress ion in elderly people aged 55 and over in northern China. A study in China showed that a vegetable-based diet was independently associated with a higher prevalence of depressive symptoms in older Chinese men (14). but plant-based eating patterns (vegan and vegetarian) were generally considered “healthy”, and has a wide range of health benefits (15). Moreover, fruits and vegetables as anti-inflammatory foods can reduce the inflammatory state to a certain extent and have a certain protective effect on depression (16). Therefore, the current study used different statistical methods to explore the relationship between different dietary patterns and the risk of depressive symptoms, taking into account whether there are some differences between men and women.

## Methods

### Study Participants

Participants in the present study were derived from the baseline of the Community Cohort Study of Nervous System Diseases (CCSNSD), an ongoing longitudinal study established by the project in 2018–2019, focusing on potential factors related to the risk of three neurological diseases, including epilepsy in patients >1-year-old and Alzheimer's disease (AD), Parkinson's disease (PD) in people ≥55 years old. In Hebei Province from May to August 2018, 2 city sites and 2 county town sites were selected in the province, 1 urban neighborhood committee and 1 suburban village were selected for each city site, and 1 county town neighborhood committee and 1 suburban village were selected for each county site. Thirty-two survey sites in 4 project provinces, each survey site is randomly selected on a household basis, and all resident family members in each selected family who meet the selection criteria are the survey objects. The questionnaire includes general demographic information, lifestyle, disease history and other information of the respondents. A food frequency scale was used to ask respondents about the frequency and amount of food/food groups consumed in the past 12 months. The frequency of intake of food/food groups was divided into: daily, weekly, monthly, yearly or never. The intake is the birth weight of the edible portion of the food consumed by the respondents each time. The project is undertaken by the Institute of Nutrition and Health of the Chinese Center for Disease Control and Prevention, in cooperation with the Center for Disease Control and Prevention. The project applies a multistage random cluster sampling method to extract samples. The protocol of this study was reviewed and approved by the Institutional Review Board of the National Institute for Nutrition and Health (No. 2017020, November 6, 2017). In addition, written informed consent was obtained for each participant before the survey.

**Figure 1:**
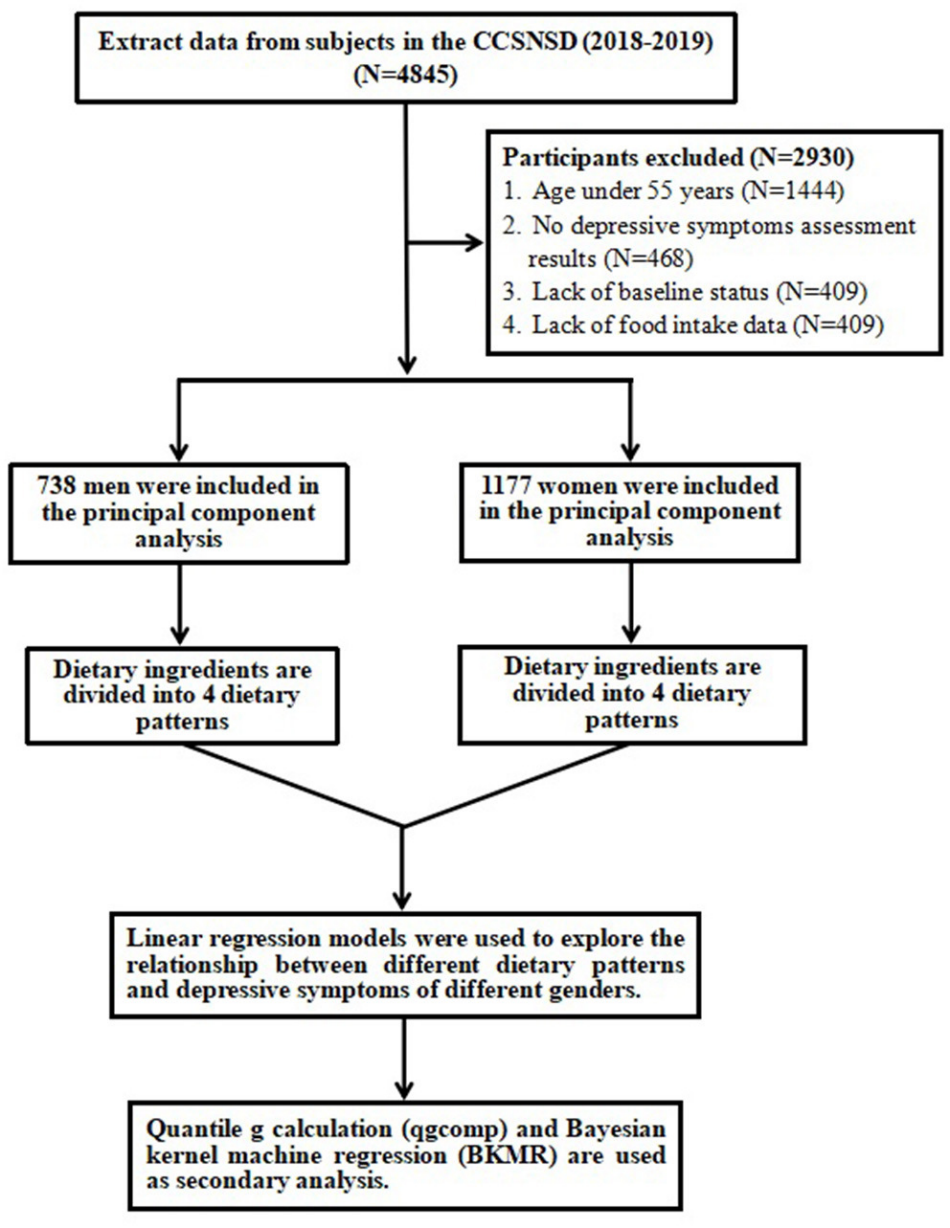
Selection process of subjects.

Among the subjects recruited in the CCSNSD cohort, the samples eligible for inclusion were (1) 55 years old and older, (2) resident population living in the sampled community, (3) absence of clinically diagnosed depressive symptoms, (4) be able to perform a normal depressive symptoms assessment, (5) completed data of sociodemographic characteristics, disease history, and food frequency questionnaire (FFQ). We excluded subjects because of (1) no depressive symptoms assessment results, (2) lack of baseline status such as education and physical activities, (3) Lack of food intake data. Finally, a total of 1,915 participants were involved in the analysis (Figure 1).

In this study, we implemented 11 dietary factors of men and women (cereals and potatoes, beans and soy products, dairy products, poultry, livestock, fish and shrimp, eggs, nuts, snacks, fresh vegetables, and fresh fruits). Principal component analysis (PCA). Four dietary patterns (PCA1, PCA2, PCA3, PCA4) are derived from male dietary factors, and 4 dietary patterns (PCA1, PCA2, PCA3, PCA4) are derived from female dietary factors analysis. Linear regression models were used to explore the relationship between different dietary patterns and depressive symptoms of men and women. We used weighted quantile sum (WQS) regression, quantile g calculation (qgcomp) and Bayesian kernel machine regression (BKMR) as the secondary analysis.

### Exposure and Outcome Variables

Dietary consumptions were assessed by a validated semi-quantitative FFQ (19). For each item, participants were asked to specify how often they consumed food or beverages on average in the previous year. The frequency of consumption response is divided into never, <1 time/month, 1–3 times/month, 1 time/week, 2–4 times/week, 5–6 times/week, 1 time/day, 2–3 times/day, 4–5 times/day, ≥6 times/day. Energy intake is calculated by multiplying the frequency of consumption per unit of food by the energy content of a specified serving. Evaluate the composition value of energy by using the Chinese food content database. In men and women, the 11 main food groups and items (cereals and potatoes, beans and soy products, diary, poultry, livestock, fish and shrimp, eggs, nuts, snacks, fresh vegetables, fresh fruits) were divided into different eating patterns using principal component analysis. Then, we used the quantile g calculation to test the combined effects of different dietary patterns on depressive symptoms in men and women. According to the standards established by the Chinese Obesity Working Group, general obesity is defined as BMI ≥ 28.0 kg/m^2^ (20).

We defined depressive symptoms according to the Geriatric Depression Scale (GDS), one of the most widely used scales to assess depressive symptoms in older adults. It consists of 30 self-assessment items with yes/no response options. A score of 0–10 indicates no depressive symptoms, a score of 11–20 indicates mild depressive symptoms, and a score of 21–30 indicates severe depressive symptoms.

### Weighted Quantile Sum Regression

WQS regression is an application of a new statistical method for high-dimensional mixture analysis. This method uses dimensionality reduction, model selection, and integrated random subset methods to provide robust parameter estimates in high-dimensional mixtures. At the same time, it can maintain robust and stable performance under different collinearity and predictor set size conditions (21).

WQS regression formula:


$$\begin{array}{l} g\left(\mu_{i}\right)=\beta_{0}+\beta_{1}\left(\overset{c}{\underset{j=1}{\sum }}w_{j}q_{ji}\right)+z_{i}^{\prime }\phi \end{array}$$

For j = 1 to *c* exposure components, q_*ji*_ is the quantile of component *j* for the *i*-th individual. The weight *w*_*j*_ is estimated for each of the *j* components, where the weight takes a value between 0 and 1 and the sum is 1. WQS regression analysis is performed in multiple steps. First, the estimation of weights uses a non-linear modeling algorithm, in which regression coefficients and weights are estimated at the same time. The overall effect of the mixture (WQS index) is estimated by β_1_, with weight is limited to a single direction, and the generalized linear model is used to connect with the average result μ_i_, along with the intercept β_0_, matrix of covariates $z_{i}^{\prime }$ and their corresponding coefficients ϕ. In the non-linear estimation step, through separate constraint analysis, the exponents in the positive and negative directions can be estimated. The advantage of concentrating inference on a single direction constraint (combined with a constraint that the sum of weights is 1) is that due to the complex correlation patterns in the quantized components, the fine-tuning of the estimate is improved. The integration step provides the advantage of stabilizing the weights while accommodating the variability of their estimates. Ideally, WQS uses a training set for model fitting in the integration step and performs hypothesis testing on the WQS index in the retention or validation set. Finally, when two indicators are estimated using a positive constraint and a negative constraint, they can be combined in the final model to evaluate their joint relationship with the average response (22).

### Quantile g-Computation

Quantile g calculation is a parameter-based, generalized linear model-based g calculation implementation to estimate the result change of a quantile while increasing all exposures in a specific mixture. We call it “mixed exposure-response”. This approach combines the inferential simplicity of weighted quantile sum (WQS) regression with the flexibility of g-computation, a method of causal effect estimation (23). Quantile g-computation does not require a “directional homogeneity” assumption that all exposures are related to the results in the same direction. In addition, this method usually allows a flexible polynomial function of mixed exposure-response, although for the sake of simplicity, we describe the implementation under the linear assumption. This model is achieved by classifying all dietary patterns into quartiles, $X_{j}^{q}$, coded as 0, 1, 2, and 3, and fitting a linear model $Y_{i}=\beta_{0}+\psi \sum_{j=1}^{p}w_{j}X_{j}^{q}+\varepsilon_{i}=\beta_{0}+\sum_{j=1}^{p}\beta_{j}X_{j}^{q}+\varepsilon_{i}$ where $\sum_{j=1}^{p}\beta_{j}=\psi$ and each dietary pattern is given a negative or positive weight. A linear model is used to continuously process the quartile values. The estimated quantile g of the exposure response ψ is the sum of the regression coefficients of the included exposures. If all β_*j*_ are in the same direction, the weight of the mixed component *k* (e.g., a specific dietary pattern) is defined as $w_{k}=\beta_{k}/\sum_{j=1}^{p}\beta_{j}$ which is the ratio of the effects produced by this ingredient, and the total is 1.0. If the dietary patterns have different influence directions, the weight is interpreted as the positive (or negative) part of the influence, and the sum of the positive and negative weights is 2.0 (24).

### Bayesian Kernel Machine Regression

In recent years, people have proposed a new hybrid health effect estimation method-Bayesian Kernel Machine Regression (BKMR). This method estimates the multivariate exposure-response function flexibly and concisely, selects variables for (possibly high-dimensional) exposure vectors and allows the use of grouping variable selection methods that can accommodate highly correlated exposures (25).

BKMR regression formula:


$$\begin{array}{l} Y_{i}=h\left(PCA1_{i},PCA2_{i},PCA3_{i},PCA4_{i}\right)+\beta^{T}C_{i}+s\left(date_{i},k\right)+\varepsilon_{i} \end{array}$$

The function h() is modeled using the Gaussian kernel exposure-response machine function, which allows interaction terms to be included. The coefficient β^*T*^ is the estimated value of the effect of the *C*th covariate for the *i*th individual. The function s() represents the natural splines for date of visit, using *k*knots as described above. The residual is represented by ε_i_. Intuitively, the Gaussian kernel assumes that two subjects with similar exposure characteristics will have more similar risk characteristic of depressive symptoms, and this similarity is the use of the kernel function. The method handles this complexity by using a kernel exposure-response machine representation for h(). We used non-informative priors for all model parameters (26).

Once fitted, summary statistics of the model for the estimation of the exposure-response function and the confidence interval (CI) for each dietary pattern, the overall link between the total levels of dietary patterns and the result, and the interaction between each dietary pattern pair are provided. We ran 8 Bayesian kernel machine regression models, including 4 dietary patterns for men (PCA1, PCA2, PCA3, PCA4) and 4 dietary patterns for women (PCA1, PCA2, PCA3, PCA4) as a result of depressive symptom. All covariates have been adjusted in 8 models.

### Covariates

We adjusted for variables previously identified as potential confounders in the literature. The included baseline characteristics include self-reported age (years), daily energy intake (kcal), education level (illiterate, elementary school, junior high school and above), employment status (yes or no); lifestyle and health-related variables include smoking (yes or No), drinking (yes or no), physical activity (yes or no), diabetes (yes or no), hypertension (yes or no), residence (urban or rural). The missing values of all variables are <5%, so the median and mode of quantitative and qualitative variables are used for inference.

### Statistical Analysis

Continuous variables are expressed as mean ± standard deviation or median (interquartile range), and the number of categorical variables (percentage) is expressed. Continuous variables, where appropriate, were compared by independent samples, unpaired, 2-sided *t*-test, or the Mann-Whitney U test. Categorical variables were compared by the chi-squared test or Fisher's exact test.

Principal component analysis (PCA) is used to determine eating patterns based on the 11 food groups mentioned above. The data adequacy of factor analysis has been evaluated by the Kaiser-Meyer-Olkin sample adequacy measure and the Bartlett ball test. Through orthogonal transformation (maximum variance rotation) rotation factor minimizes the correlation between them and improves their interpretability. After evaluating eigenvalues, gravel plots, factor interpretability, and variance interpretation, a five-factor solution was selected. If the absolute correlation (factor loading) between the food group and the model is ≤ 0.20, the food group is considered to have made a significant contribution to the model. The factor score for each dietary pattern of each participant was calculated by weighting the intake of the food group according to their factor load. The labeling of eating patterns is based on the interpretation of the main food groups with high factor loads in each eating pattern.

We used logistic regression models to evaluate the association between various diet patterns and obesity. Calculate the tolerance and variance inflation factor (VIF) values to evaluate the multicollinearity between the covariates. Tolerance <0.1 and VIF > 10 are considered to indicate multicollinearity. All models were adjusted for maternal age, BMI, education level, employment status, smoking, drinking, physical activity, diabetes, hypertension, and residence.

Weighted Quantile Sum Regression, Quantile g-computation and Bayesian Kernel Machine Regression (BKMR) are used to estimate the individual and overall effects of dietary patterns on the risk of depression in the elderly. BKMR is a semi-parametric method that can be used to estimate the effects of individual mixed dietary pattern components, the overall mixed dietary pattern effects, and the interaction between the mixed dietary pattern components. The probabilistic link function is used to fit the exposure-response relationship of the binary outcome (i.e., depression). As mentioned above, the BKMR model is adjusted for the corresponding covariates. The overall mixed effect of eating patterns is estimated by subtracting the average of all eating pattern scores at the 75th percentile from the average of the eating pattern scores at the 25th percentile. Similarly, by subtracting the average result value when the designated dietary pattern score is in the 25th percentile and the average result when the designated dietary pattern score is in the 75th percentile, the individual influence of each dietary pattern score mixture component is estimated. The median scores of other dietary patterns were fixed, and all covariates remained the same. Plotting is used to assess the overall association between the dietary pattern score mixture and the risk of depression, and to visualize the exposure-response relationship of each dietary pattern score, while keeping other exposures at the 50th percentile and covariates unchanged. All BKMR models was fit 50,000 iterations using the Markov Chain Monte Carlo (MCMC) sampler.

Although BKMR benefits from the flexibility of modeling exposure-response functions and allows for non-linear and non-additive associations between multiple dietary pattern scores and depression risk, it cannot provide concise parameter inference. We further applied the recently developed quantile-based g calculation method to explore the joint effect based on parameter inference. This method combines the simplicity of reasoning of weighted quantile regression (WQS) with the flexibility of g calculation, and also allows the non-linearity and non-additiveness of BKMR, which seems smaller and more robust. We use this method to simulate the combined and individual effects of exposure to all dietary patterns on the risk of depression. The binomial distribution is designated as the link function. The parameter q represents the specified quantile and is set to 4 because most dose-response relationships do not show a significant non-linear pattern. Five hundred boot iterations were performed to calculate CIs.

All statistical analyses were conducted in R (version 3.6.1, R Development Core Team 2019). The BKMR model, qgcomp model were implemented using the R package “bkmr,” “qgcomp,” respectively. An α level of 5% (two-sided) was considered statistically significant.

## Results

### Population Characteristics

The study included 1,915 elderly participants over 55 years of age, of whom 465 (24.3%) were diagnosed with depressive symptoms. The demographic characteristics of the participants are shown in Table 1. Compared with non-depressed residents, the majority of depressed residents are women, and more people live in urban areas and have fewer physical activities. Comparing the differences in baseline characteristics between men and women at the same time, the results show that compared with women, men have a higher daily energy intake, at the same time have a higher proportion of smoking and drinking, and less physical activity (Table 2).

**Table 1:** Characteristics of study participants of men and women.

**Characteristic**	**Frequency (%) or median (IQR)**		
	**Male (***n*** = 738)**	**Female (***n*** = 1,177)**	***p*** **-value**
**Age (years)**			0.154
	64 (61–70)	65 (61–70.5)	
**Daily energy intake (kcal/day)**			<0.01
	1,330.2 (1,217.3–1,573.5)	1,257.7 (1,044.2–1,584.1)	
**BMI (kg/m** ^**2**^ **)**			<0.01
BMI <18.5 (Underweight)	19 (2.6)	35 (3.0)	
18.5 ≤ BMI <24.00 (normal weight)	293 (39.7)	441 (37.5)	
24.00 ≤ BMI <28.00 (overweight)	321 (43.5)	437 (37.1)	
BMI ≥ 28.00 (obese)	105 (14.2)	264 (22.4)	
**Employment**			<0.01
No	552 (74.8)	1,096 (93.1)	
Yes	186 (25.2)	81 (6.9)	
**Education level**			<0.01
Illiteracy	117 (15.9)	361 (30.7)	
Primary school	224 (30.3)	393 (33.4)	
Junior high school/above	397 (53.8)	423 (35.9)	
**Tobacco smoking**			<0.01
No	490 (66.4)	1,121 (95.2)	
Yes	248 (33.6)	56 (4.8)	
**Alcohol drinking**			<0.01
No	580 (78.6)	1,153 (98.0)	
Yes	158 (21.4)	24 (2.0)	
**Diabetes**			0.97
No	619 (83.9)	988 (83.9)	
Yes	119 (16.1)	189 (16.1)	
**Physical activity**			<0.01
No	502 (68.0)	670 (56.9)	
Yes	236 (32.0)	507 (43.1)	
**Hypertension**			0.29
No	283 (38.3)	480 (40.8)	
Yes	455 (61.7)	697 (59.2)	
**Residence**			0.01
Urban	181 (24.5)	359 (30.5)	
Rural	557 (75.5)	818 (69.5)	

**Table 2:** The distribution of baseline characteristics between study participants who are depressed or not.

**Characteristic**	**Frequency (%) or median (IQR)**		
	**Non-depression (***n*** = 1,450)**	**Depression (***n*** = 465)**	***p*** **-value**
**Age (years)**			0.57
	65.0 (61.0–70.0)	65.0 (61.0–70.0)	
**Daily energy intake (kcal/day)**			0.80
	1,321.5 (1097.1–1,573.3)	1283.4 (1,119.9–1,600.4)	
**Gender**			<0.01
Male	591 (40.8)	147 (31.6)	
Female	859 (59.2)	318 (68.4)	
**BMI (kg/m** ^**2**^ **)**			0.29
BMI <18.5 (Underweight)	39 (2.7)	15 (3.2)	
18.5 ≤ BMI <24.00 (normal weight)	568 (39.2)	166 (35.7)	
24.00 ≤ B MI <28.00 (overweight)	576 (39.7)	182 (39.1)	
BMI ≥ 28.00 (obese)	267 (18.4)	102 (21.9)	
**Employment**			0.02
No	1,235 (85.2)	413 (88.8)	
Yes	215 (14.8)	52 (11.2)	
**Education level**			0.38
Illiteracy	373 (25.7)	105 (22.6)	
Primary school	465 (32.1)	152 (32.7)	
Junior high school/above	612 (42.2)	208 (44.7)	
**Tobacco smoking**			0.98
No	1,220 (84.1)	391 (84.1)	
Yes	230 (15.9)	74 (15.9)	
**Alcohol drinking**			0.45
No	1,308 (90.2)	425 (91.4)	
Yes	142 (9.8)	40 (8.6)	
**Diabetes**			0.64
No	1,220 (84.1)	387 (83.2)	
Yes	230 (15.9)	78 (16.8)	
**Physical activity**			<0.01
No	702 (48.4)	304 (65.4)	
Yes	748 (51.6)	161 (34.6)	
**Hypertension**			0.12
No	592 (40.8)	171 (36.8)	
Yes	858 (59.2)	294 (63.2)	
**Residence**			0.01
Urban	392 (27.0)	148 (31.8)	
Rural	1,058 (73.0)	317 (68.2)	

Table 3 shows the distribution of intake levels of 11 food types among men and women. There were significant differences in the intake levels of cereals, potatoes, livestock and milk between men and women (*P* < 0.05), and there was no significant difference in intake levels of other food types.

**Table 3:** Distribution of food groups between men and women.

**Food groups**	**Median (IQR)**		
	**Male (***n*** = 738)**	**Female (***n*** = 1,177)**	***p*** **-value**
Cereals and potatoes (g)	330.45 (237.09–450.00)	292.86 (207.69–385.35)	<0.001
Beans and soy products (g)	25.46 (6.07–59.02)	28.57 (6.89–63.44)	0.168
Poultry (g)	3.93 (0.00–13.11)	3.28 (0.00–12.99)	0.983
Livestock (g)	28.57 (14.29–57.14)	27.92 (10.40–48.38)	0.001
Fish and shrimp (g)	4.92 (0.00–14.29)	3.50 (0.00–12.66)	0.133
Eggs (g)	60.00 (33.78–63.93)	60.00 (30.11–63.36)	0.792
Nuts (g)	0.00 (0.00–3.28)	0.00 (0.00–3.08)	0.215
Snacks (g)	0.00 (0.00–3.10)	0.00 (0.00–4.29)	0.100
Fresh vegetables (g)	161.34 (78.51–312.10)	171.43 (85.95–308.04)	0.513
Fresh fruits (g)	53.59 (17.00–114.29)	57.14 (21.04–117.35)	0.174
Dairy (ml)	0.00 (0.00–37.03)	0.00 (0.00–100.00)	<0.001

This study passed the Kaiser-Meyer-Olkin test, and the sampling adequacy value of the 11 food categories for food group intake is 0.801, which is significantly different from the Bartlett sphere test (*P* < 0.001), indicating that these 11 food categories were suitable for principal component analysis. After each maximum rotation, the principal component analysis of men and women revealed the influence of four similar main eating patterns and table patterns. The four patterns of men accounted for 32.6% of the total changes in dietary pattern, and the four patterns of women accounted for 36.8% of the total changes in dietary pattern. The diet pattern is as follows: principal component 1 is defined as a vegetable and fruit diet pattern; principal component 2 is defined as a grain and potato diet pattern; principal component 3 is defined as a meat diet pattern; principal component 4 is defined as an egg-milk diet pattern (Table 4).

**Table 4:** Factor load matrix of dietary pattern between men and women.

**Food groups**	**Male (*****n*** **= 738)**				**Female (*****n*** **= 1,177)**			
	**PCA1**	**PCA2**	**PCA3**	**PCA4**	**PCA1**	**PCA2**	**PCA3**	**PCA4**
Cereals and potatoes (g)	−0.004	0.019	0.93	−0.014	−0.019	−0.027	0.854	0.012
Beans and soy products (g)	0.412	0.108	−0.089	0.035	0.487	0.264	−0.057	−0.082
Poultry (g)	0.017	0.759	0.164	−0.008	0.098	0.778	−0.002	−0.006
Livestock (g)	0.194	0.553	−0.034	−0.037	0.122	0.612	−0.174	0.427
Fish and shrimp (g)	0.303	0.561	−0.168	−0.201	0.283	−0.004	−0.579	0.084
Eggs (g)	0.069	0.001	−0.024	0.759	0.025	0.061	0.502	0.864
Nuts (g)	−0.053	0.554	0.017	0.458	−0.009	0.451	0.261	−0.265
Snacks (g)	0.095	−0.096	−0.013	0.842	0.27	−0.048	0.545	−0.023
Fresh vegetables (g)	0.767	0.224	−0.005	−0.039	0.755	0.149	−0.114	0.08
Fresh fruits (g)	0.663	0.069	0.381	−0.019	0.674	0.08	0.274	−0.065
Dairy (ml)	0.555	−0.039	−0.084	0.622	0.687	−0.146	−0.005	0.682

Table 5 shows the relationship between different diet patterns and obesity for men and women. The results showed that among men, the meat-based diet was significantly positively correlated with obesity, while the egg-milk-based diet was negatively correlated with obesity. Similarly, we found that among women, a diet based on fruits and vegetables is negatively correlated with obesity, while a diet based on cereals and potatoes and meat is positively correlated with obesity. The WQS regression results further supported the regression results (Table 6).

**Table 5:** Different gender dietary patterns are associated with the risk of depression.

**Factor**	**Male**		**Female**	
	**OR (95%CI)**	***p*** **-value**	**OR (95%CI)**	***p*** **-value**
Factor 1	0.86 (0.62–1.06)	0.182	0.76 (0.58–0.96)	0.026
Factor 2	1.16 (1.05–1.31)	0.015	1.10 (1.02–1.28)	0.008
Factor 3	1.12 (1.08–1.36)	0.027	1.18 (1.02–1.41)	0.036
Factor 4	0.85 (0.69–0.97)	0.041	0.96 (0.81–1.08)	0.126

**Table 6:** Associations of WQS index with depression in both sexes.

**Gender**	**WQS direction**	**Depression**
		**OR (95%CI)**
Male (*n* = 738)	*N*	0.92 (0.86–1.08)
	*P*	1.12 (1.05–1.28)
Female (*n* = 1,177)	*N*	0.85 (0.76–1.08)
	*P*	1.08 (1.01–1.22)

**Figure 2:**
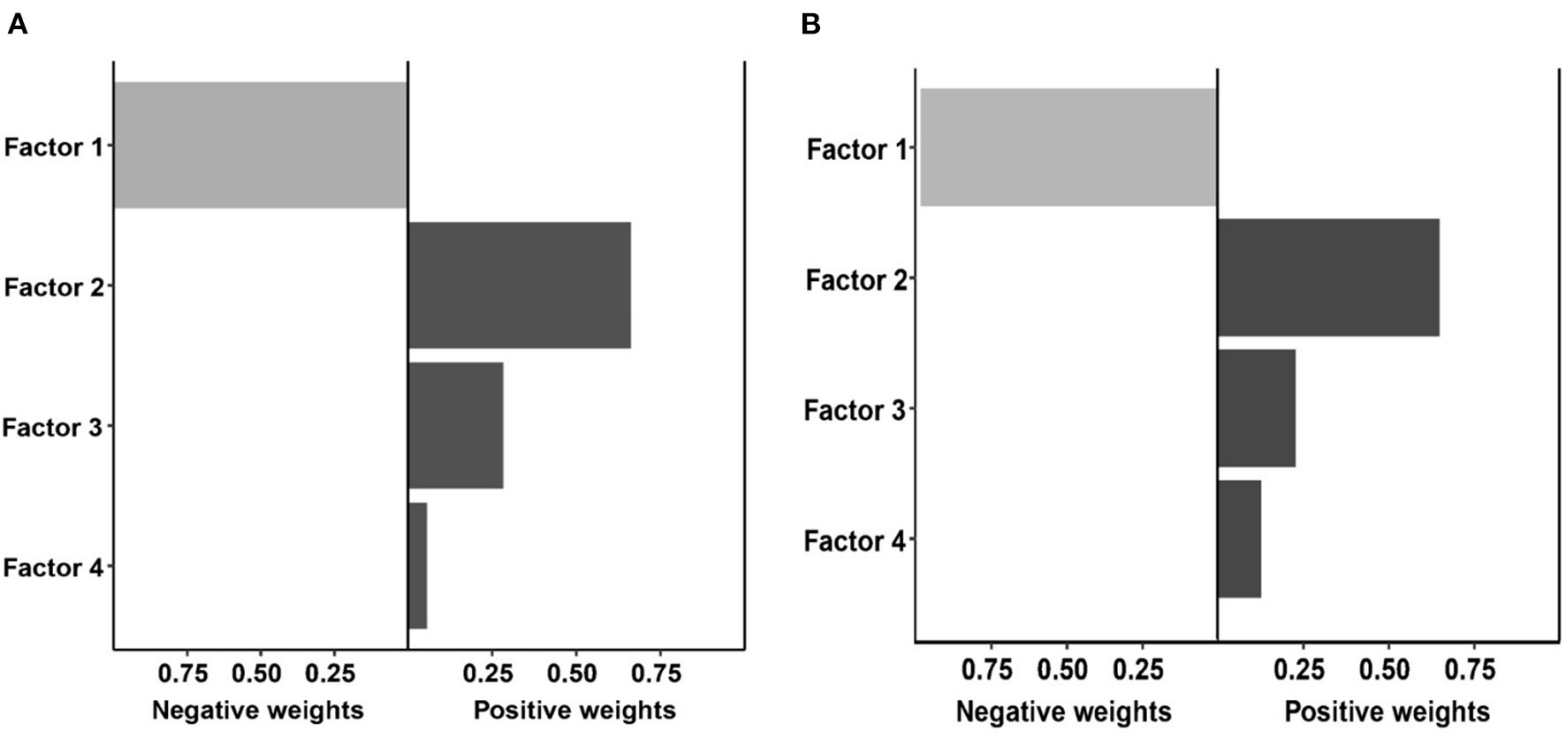
Weights representing the proportion of the positive or negative partial effect for each diet pattern in the quantile g-computation model with **(A)** the male group, and **(B)** the female group.

**Figure 3:**
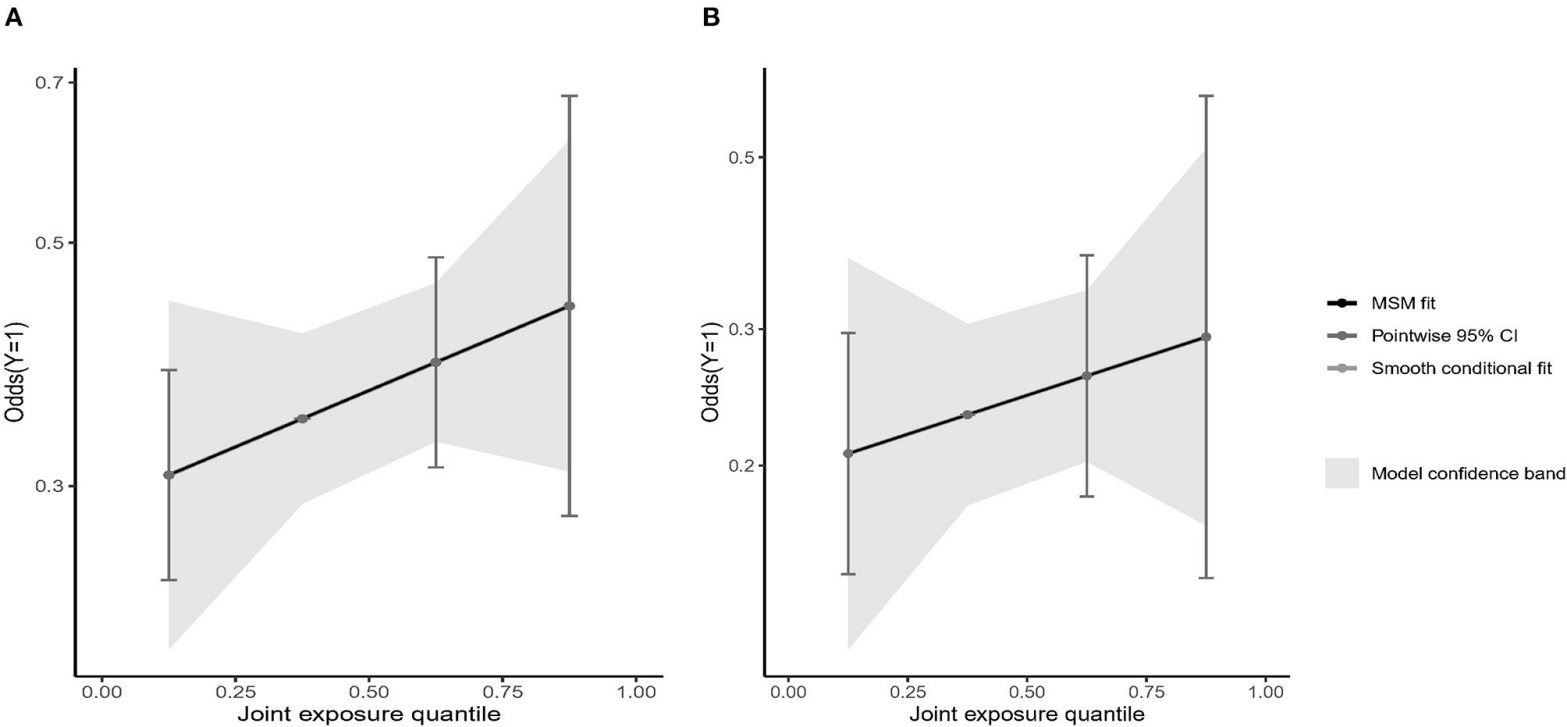
Weights representing the proportion of the positive or negative overall effect for diet pattern in the quantile g-computation model with **(A)** the male group, and **(B)** the female group.

The results of the qgcomp mixed exposure model analysis showed that for the entire male population, it was observed to be positively correlated with the subsequent risk of depression [OR (95%CI): 1.12 (1.06–1.26)]; in women, the overall impact of dietary patterns was positively correlated with the risk of depression [OR (95%CI): 1.06 (1.02–1.20)]. The weight of each diet pattern in the population and gender group is shown in Figures 2, 3. Among the positively correlated food types, the meat-based diet has the largest weight among men and women. Among them, the meat-based diet has a significantly higher positive effect on the risk of depression in men than in women, while the vegetable and fruit-based diet. The pattern shows a significant negative correlation in both men and women, and the negative effect in women is significantly higher than that in men.

**Figure 4:**
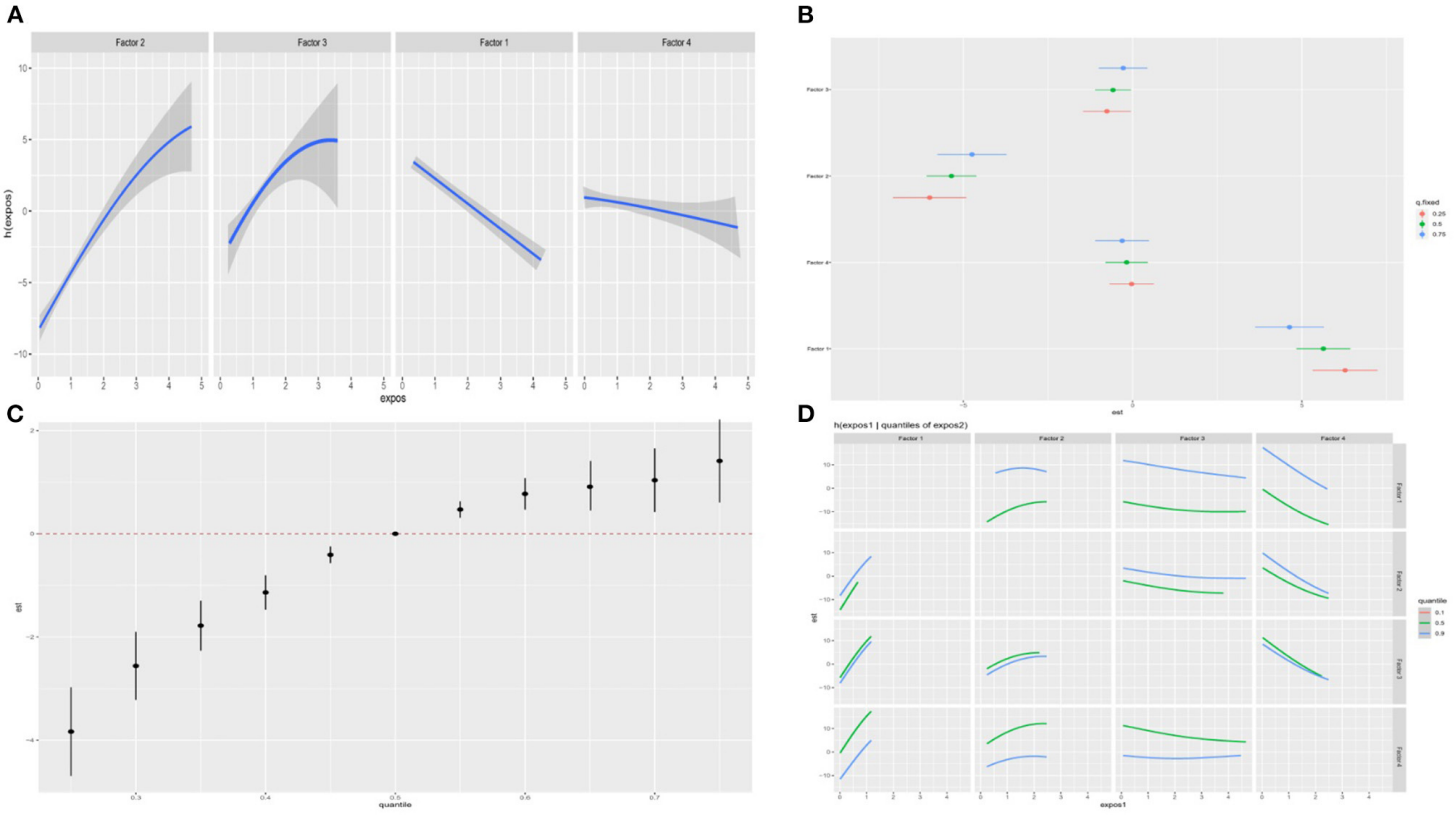
The BKMR model is used to study the association between dietary patterns among men and depression in the elderly. The model is based on age, gender, education level, employment status, smoking status, drinking status, daily energy intake, hypertension and diabetes, and dietary patterns. **(A)** The single dietary pattern effect (estimates and 95% credible intervals). **(B)** The univariate nutrient response function of each dietary pattern has a 95% confidence band, and the other nutrients are fixed at the median. **(C)** Cumulative effects of dietary patterns (estimated value and 95% confidence interval). Compared with other nutrients in the 50th percentile, the nutrients are in a specific percentile (X-axis). **(D)** Bivariate exposure response function of each two dietary patterns in depression.

**Figure 5:**
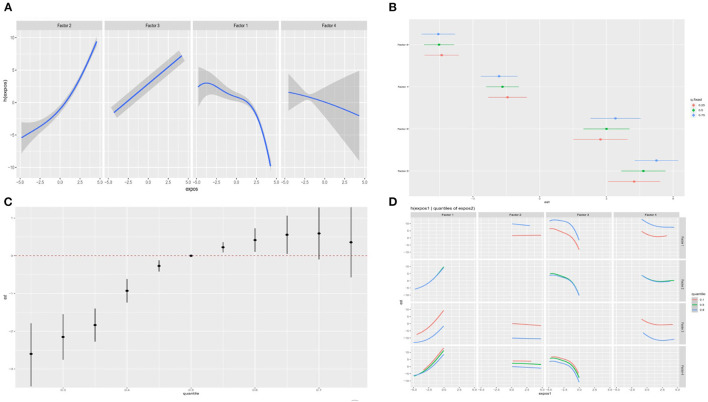
The BKMR model is used to study the association between dietary patterns among women and depression in the elderly. The model is based on age, gender, education level, employment status, smoking status, drinking status, daily energy intake, hypertension and diabetes, and dietary patterns. **(A)** The single dietary pattern effect (estimates and 95% credible intervals). **(B)** The univariate nutrient response function of each dietary pattern has a 95% confidence band, and the other nutrients are fixed at the median. **(C)** Cumulative effects of dietary patterns (estimated value and 95% confidence interval). Compared with other nutrients in the 50th percentile, the nutrients are in a specific percentile (X-axis). **(D)** Bivariate exposure response function of each two dietary patterns in depression.

We showed the visualization of the BKMR model. First of all, we discovered the cumulative effect of mixed eating patterns in our research. We can see that whether it is a male or a female, the risk of depression increases as the percentile of the principal component mixed effect increases. The overall effect was statistically significant (*P* < 0.05; Figures 4A, 5A). Then, we tried to find the single effect of dietary pattern scores by estimating univariate summaries of changes in depression risk associated with changes in single dietary pattern scores. We found that men's meat eating pattern scores and depression risk showed more significant positive effects than women. Compared with men, women's fruit and vegetable eating pattern scores and depression risk showed more significant negative effects. At the same time, cereal and potato diet patterns have a negative impact on the risk of depression, but the egg-milk diet pattern showed similar weak positive effects in the two groups (Figures 4B, 5B).

In order to study the potential non-linearity of the exposure-response function, we then estimated the univariate relationship between each dietary pattern score and the elderly's depression risk, and fixed all remaining dietary pattern scores at the 50th percentile. The figure shows that both men and women found that the meat diet pattern score significantly increased the risk of depression, while the egg-milk diet pattern and the fresh fruit and vegetable diet pattern score significantly reduced the risk of depression. It is worth noting that scores of grain and potato diet patterns can reduce the risk of depression in women, while men tend to increase the risk. The egg-milk diet scores of the two groups were significantly negatively correlated with the risk of depression (Figures 4C, 5C). To further explore the potential relationship between dietary pattern scores, we plotted a bivariate cross-section of the exposure-response function. Figures 4D, 5D, respectively, show the difference in depression risk as a response variable of male and female eating pattern scores (the dietary pattern score is fixed in the middle number). The parallel exposure-response relationship showed no evidence of interaction between the eating pattern scores.

## Discussion

In this study, we used a variety of statistical models to explore the association between different gender and different eating patterns and depression in the elderly. In general, mixed exposure regardless of male and female dietary patterns may increase the risk of depression. As for specific dietary patterns, meat diet patterns significantly increase the risk of depression in the elderly, and vegetable and fruit diet patterns are negatively correlated with the risk of depression in the elderly. Compared with men, women's intake of egg and milk-based diet can significantly reduce the risk of depression. At the same time, no significant interaction between the various diet patterns was observed in both men and women.

One of the dietary patterns in the results of this study is associated with an increased risk of depression, and this diet is a dietary ingredient similar to the “Western-style” dietary patterns from various other studies. This is consistent with some previous conclusions that reported a positive correlation between Western dietary patterns and depression (27, 28). However, a recent systematic review reported that there is no statistically significant link between Western diet and depression (29). The inconsistencies observed in the above studies may be due to the heterogeneity of diet and depression assessment methods, as well as some major differences in the basic characteristics and dietary intake of research subjects from different cultural backgrounds. The negative impact of this model may be based on the synergistic combination of the aforementioned unhealthy ingredients. There are several possible explanations for the harmful effects of this model. First, red meat rich in saturated fatty acids may be related to cardiovascular disease by promoting insulin sensitivity, and cardiovascular disease may be involved in the pathogenesis of depression (30, 31). In addition, regular consumption of red meat is associated with high levels of low-grade inflammation, and low-grade inflammation is positively correlated with depression (32). A study conducted in 1,046 Australian women showed that there was a significant positive correlation between the consumption of red meat and major depression and anxiety (33). Finally, sugar-sweetened beverages usually contain a lot of sugar, which changes the state of oxidative stress (34). A study in China showed that regular consumption of soft drinks may increase the risk of depression (35).

We have also observed that a diet with more rice, dark vegetables, and light vegetables may reduce the risk of depression. A recent systematic review of observational studies described that adhering to a healthy diet and eating more fruits, vegetables, fish, and whole grains are associated with lowering the risk of depression (36). A cross-sectional study conducted in Japan reported a negative correlation between a healthy Japanese diet pattern and the risk of depression. This diet pattern is characterized by a large intake of vegetables, fruits, soy products, and mushrooms (37). The protective effect of this model may be partly attributable to its regular consumption of vegetables. Reasonable mechanisms for this connection from the vegetable perspective are as follows: First, oxidative stress may be related to the abnormalities of the amygdala and hippocampus in patients with severe depression (38). Vegetables contain a lot of antioxidant compounds, which are beneficial for depression by reducing oxidative stress. Oxidative stress is the biological process of excess reactive oxygen species (ROS) (30). The brain, especially the amygdala and hippocampus, is very sensitive to oxidative stress. Secondly, the anti-inflammatory properties of vegetables regulate mood and cognition by affecting the production of monoamines (39). Third, folic acid, which is needed by the neurotransmitter, may also help prevent depression, and vegetables have high levels of folic acid (40). According to reports, low folate status is associated with depression. Studies have shown that the lack of folic acid plays a key role in the pathophysiological mechanism of depression, which is achieved by increasing the concentration level of homocysteine and reducing the availability of s-adenosylmethionine (41). Finally, because there are reports that insulin resistance is a risk factor for depression, and there is evidence that insulin resistance is negatively related to high levels of fiber in the diet, the beneficial effects of vegetables may be partly attributed to dietary fiber (30, 42).

Only five other prospective studies have analyzed the role of dietary patterns in depression. By using the “a priori” approach, in the SUN cohort, higher adherence to the Mediterranean diet was associated with a lower risk of depression (43). Akbaraly et al. used PCA among participants in the Whitehall II cohort to point out that compared with the lowest tertile, people in the highest tertile of processed foods had a higher risk of depressive symptoms [multivariate odds ratio (OR) = 1.69; 95% CI, 1.10, 2.60] (44). Using PCA among participants in ALSWH, Rienks et al. observed that the incidence of depressive symptoms was lower in the bottom quintile compared with the top quintile of women with a Mediterranean diet (multivariate OR = 0.63; 95% CI, 0.47, 0.85) (45). After excluding participants with significant depressive symptoms at baseline, Le Port et al. did not observe a significant association between health patterns and depressive symptoms, but the protective effect of the traditional model was still significant. Among NHS participants, we recently analyzed the relationship between PCA-defined dietary patterns (cautious and Western patterns) and depression risk (46).

Although the secretion of pro-inflammatory cytokines increases in depression, exposure to cytokines induces depression symptoms, and some antidepressants have anti-inflammatory properties, it is not clear whether the relationship between inflammation and depression is the cause (47). However, a meta-analysis suggests that the relationship between inflammation and depression may be two-way (48). The etiology of depression is complex, involving multiple factors, and multiple mechanisms have been proposed. Cytokines may promote the development of depression through several pathophysiological mechanisms (49). Inflammation is a common link in many chronic diseases (such as cardiovascular disease, obesity, diabetes, and cancer), which are also risk factors for depression. Diet may stimulate chronic inflammatory diseases, and we have found that some foods are related to inflammation. In fact, we have previously identified a similar pro-inflammatory diet pattern, which is related to the increased risk of diabetes in the NHS and NHS2. Refined grains and sugary soft drinks that are associated with obesity and diabetes risk have an important effect on blood glucose load and may increase the susceptibility to the development of chronic inflammation. Red meat is associated with biomarkers of inflammation and increases the risk of diabetes, cardiovascular disease and cancer mortality, and depression (50). In the previous analysis of NHS data, we did not find any significant relationship between the intake of fish, fatty fish, and long-chain omega-3 and depression. These findings are also consistent with a recently published meta-analysis of 13 randomized, double-blind, placebo-controlled trials, which concluded that long-chain omega-3 supplements have no statistically and clinical significance for the severity of depressive symptoms (51, 52). In addition, a recent systematic review showed that long-chain omega-3 (0.9–2 g/day) does not change the inflammation biomarkers in healthy subjects (53). A healthy diet is consistent with current dietary guidelines, recommending high intake of fruits, vegetables, whole grains, poultry, fish, and low-fat dairy products.

Our findings showed that a fruit- and vegetable-based dietary pattern is associated with a healthier and lower risk of depression, while a meat-based dietary pattern is associated with a higher risk of depression, an associative effect that varies by gender. Therefore, while advocating to increase the consumption of fruits and vegetables, the influence of gender differences should be considered. These results will inform, in part, the implementation of dietary guidelines and public health efforts to adjust dietary recommendations. Whereas, since our study is based on baseline data from a cohort study, future related studies (longitudinal and follow-up surveys) are still needed to be thoroughly conducted in large populations to generate more consistent and robust results to improve the precision of the association, And enhance the overall understanding of the potential human health risks of different dietary patterns.

Several limitations need to be considered in this study. First, in the context of a cross-sectional design, exposure and outcome are assessed simultaneously, so the causal direction of the association between diet and depression is uncertain. Future longitudinal studies are needed to confirm our findings. Second, this study did not use clinical diagnostic methods to diagnose depression, but used the GDS scale to assess depression, which only represents a rough indicator of depression. But the GDS scale has been shown to be a highly sensitive screening tool in older adults for depression. Third, in the analysis of the diet, the analysis method of the principal component method was used, which was determined in a subjective manner in determining the number of factors of the principal components to be retained. the first. Fourth, the sample size of this study is relatively small, and we have relative limitations in the analysis of statistical power. It is recommended that follow-up studies expand the sample size for further analysis.

## Conclusions

In the study population, after controlling for other baseline indicators and predictors of dietary pattern exposure, a fruit and vegetable-based diet was associated with a slightly healthier and lower risk of depression, while a meat-based dietary pattern associated with a higher risk of depression, and this association effect varies between genders. Our results suggest that a healthy eating pattern is associated with a reduced risk of depression, whereas a western/unhealthy eating pattern is associated with an increased risk of depression. This step suggests that healthy dietary pattern intervention may play a crucial role in the prevention and treatment of depression in the elderly.

## Data Availability Statement

The original contributions presented in the study are included in the article/supplementary materials, further inquiries can be directed to the corresponding author.

## Ethics Statement

The studies involving human participants were reviewed and approved by the Institutional Review Board of the National Institute for Nutrition and Health, Chinese Center for Disease Control and Prevention. The patients/participants provided their written informed consent to participate in this study.

## Author Contributions

YM and RL: had full access to all of the data in the study, take responsibility for the integrity of the data, the accuracy of the data analysis, and concept and design. YM, WZ, and LZ: drafting of the manuscript. WZ and RL: statistical analysis. ZL and XH: obtained funding. WZ, RL, and LZ: administrative, technical, or material support. YM: supervision. All authors acquisition, analysis, or interpretation of data and critical revision of the manuscript for important intellectual content. All authors contributed to the article and approved the submitted version.

## Funding

This study was supported by Community Cohort Study on Specialized Nervous System Diseases (No. 2017YFC0907701).

## Conflict of Interest

The authors declare that the research was conducted in the absence of any commercial or financial relationships that could be construed as a potential conflict of interest.

## Publisher's Note

All claims expressed in this article are solely those of the authors and do not necessarily represent those of their affiliated organizations, or those of the publisher, the editors and the reviewers. Any product that may be evaluated in this article, or claim that may be made by its manufacturer, is not guaranteed or endorsed by the publisher.
